# A physical map of the papaya genome with integrated genetic map and genome sequence

**DOI:** 10.1186/1471-2164-10-371

**Published:** 2009-08-07

**Authors:** Qingyi Yu, Eric Tong, Rachel L Skelton, John E Bowers, Meghan R Jones, Jan E Murray, Shaobin Hou, Peizhu Guan, Ricelle A Acob, Ming-Cheng Luo, Paul H Moore, Maqsudul Alam, Andrew H Paterson, Ray Ming

**Affiliations:** 1 Cellular and Molecular Biology Research Unit, Hawaii Agriculture Research Center, Aiea, HI 96701, USA; 2 Plant Genome Mapping Laboratory, University of Georgia, Athens, GA 30602, USA; 3 Department of Plant Biology, University of Illinois at Urbana-Champaign, Urbana, IL 61801, USA; 4 Center for Advanced Studies in Genomics, Proteomics and Bioinformatics, University of Hawaii, Honolulu, HI 96822, USA; 5 Department of Molecular Bioscience and Bioengineering, University of Hawaii, Honolulu, HI 96822, USA; 6 Department of Plant Sciences, University of California, Davis, CA 95616, USA; 7 USDA-ARS, Pacific Basin Agricultural Research Center, Hilo, HI 96720, USA

## Abstract

**Background:**

Papaya is a major fruit crop in tropical and subtropical regions worldwide and has primitive sex chromosomes controlling sex determination in this trioecious species. The papaya genome was recently sequenced because of its agricultural importance, unique biological features, and successful application of transgenic papaya for resistance to papaya ringspot virus. As a part of the genome sequencing project, we constructed a BAC-based physical map using a high information-content fingerprinting approach to assist whole genome shotgun sequence assembly.

**Results:**

The physical map consists of 963 contigs, representing 9.4× genome equivalents, and was integrated with the genetic map and genome sequence using BAC end sequences and a sequence-tagged high-density genetic map. The estimated genome coverage of the physical map is about 95.8%, while 72.4% of the genome was aligned to the genetic map. A total of 1,181 high quality overgo (overlapping oligonucleotide) probes representing conserved sequences in *Arabidopsis *and genetically mapped loci in *Brassica *were anchored on the physical map, which provides a foundation for comparative genomics in the Brassicales. The integrated genetic and physical map aligned with the genome sequence revealed recombination hotspots as well as regions suppressed for recombination across the genome, particularly on the recently evolved sex chromosomes. Suppression of recombination spread to the adjacent region of the male specific region of the Y chromosome (MSY), and recombination rates were recovered gradually and then exceeded the genome average. Recombination hotspots were observed at about 10 Mb away on both sides of the MSY, showing 7-fold increase compared with the genome wide average, demonstrating the dynamics of recombination of the sex chromosomes.

**Conclusion:**

A BAC-based physical map of papaya was constructed and integrated with the genetic map and genome sequence. The integrated map facilitated the draft genome assembly, and is a valuable resource for comparative genomics and map-based cloning of agronomically and economically important genes and for sex chromosome research.

## Background

Papaya, *Carica papaya *L., is a fast-growing fruit crop grown in tropical and subtropical regions worldwide. Papaya fruit is among the most nutritious available. It is rich in vitamins A and C, and has been recommended for prevention of vitamin A deficiency in tropical and subtropical developing countries [1]. In addition to fresh fruit production, papaya is grown for papain, a proteolytic enzyme widely used in food processing, cosmetic, pharmaceutical, and leather industries, as well as medical applications [2,3].

The majority of flowering plants, unlike most animal species that produce unisexual individuals, produce 'perfect' flowers that contain both male and female organs. It has been reported that only 6% of 250,000 angiosperm species are dioecious, i.e. having male and female individuals [4]. Papaya is one of the rare species classified as trioecious because its individuals exist as one of three sex types – female, male, or hermaphrodite. Sex determination in papaya is controlled by a pair of recently evolved sex chromosomes with the genotype XX for female, XY for male, and XY^h ^for hermaphrodite [5,6]. The difference between X and Y (or Y^h^) chromosomes is a small non-recombination region, which is called the male-specific region of the Y chromosome (MSY). The Y and Y^h ^originated from an ancestral Y chromosome about 73,000 years ago and share 98.6% DNA sequence identity in the MSY [7]. The physical size of the MSY is about 8 Mbp and accounts for only about 13% of the papaya Y chromosome [8,9]. Papaya is an excellent system in which to study the early events of sex chromosome evolution.

Papaya originated in Central America where it was domesticated by aboriginals in this region [10]. Hermaphrodite papaya trees are self-pollinated and hermaphrodite cultivars are in production in most papaya growing regions for the obvious reason that every hermaphrodite tree produces fruit. Nevertheless, dioecious cultivars are used in India, Australia, and South Africa to assure greater fruit production under cool winter temperatures. The first transgenic papaya cultivar SunUp is derived from hermaphrodite Solo cultivar Sunset that has undergone more than 25 generations of self pollination [10]. The genomic DNA of hermaphrodite 'SunUp', that contains the X and Y^h ^chromosomes, was used for construction of a papaya bacterial artificial chromosome (BAC) library [11], whereas a 'SunUp' female with XX chromosomes was used for whole genome shotgun sequencing to avoid the complication of assembling the heterozygous region of the X and Y^h ^chromosomes [9].

Papaya belongs to the small family Caricacea and is in the order Brassicales. Papaya and *Arabidopsis *diverged from a common ancestor about 72 million years ago. Thus, papaya can serve as an outgroup for comparative study of Brassicaceae genomes. Papaya has a small genome of 372 Mbp with nine pairs of chromosomes, a short juvenile phase of 3–8 months, and a short generation time of 9–15 months. Papaya is one of the few fruit tree crops that can flower and fruit throughout the year, providing a constant supply of flower buds and fruits. Each mature fruit contains 800–1000 seeds, and a single F1 tree could produce a large segregating F2 population for genetic studies. Clonal propagation can be done easily from cuttings or through micro-propagation. An efficient transformation system has been established as demonstrated by the success of transgenic papaya that saved the Hawaiian papaya industry [12,13]. These attributes make papaya an excellent model system for tropical fruit trees in which to study a number of biological processes, including sex chromosome evolution [5,7,8], cell wall biosynthesis and degradation [14], vegetative/reproductive growth phase transition, flower development [15,16], fruit development, fruit ripening, and post-harvesting physiology [14].

Significant progress has been made in recent years in developing genomic resources to expedite genome research in papaya. A BAC library of 'SunUp' hermaphrodite was constructed with 13.7× genome equivalents, providing the foundation for studying papaya genome structure and organization [11]. BAC ends of this library were sequenced [17], providing the first glimpse of the sequence composition of the papaya genome. Two high-density genetic linkage maps have been constructed [18,19], providing essential tools for comparative genomic analysis, marker-assisted selection, and genomic dissection of complex traits. The first high-density genetic map of papaya was constructed with 1,501 markers, including 1,498 amplified fragment length polymorphism (AFLP) markers, 2 morphological markers, and one transgenic marker [18]. Although this map has high-density, it is not suitable for aligning papaya genome sequence to linkage groups and integrating genetic and physical maps due to the anonymous nature of AFLP markers. To overcome this limitation, highly informative sequence-based simple sequence repeat (SSR) markers were used to construct the second high-density genetic map, containing 707 markers including 706 sequence-based SSR markers and one morphological marker [19]. These SSR markers were developed from either BAC end or whole-genome shotgun sequence reads [19], and this map is a crucial resource for integration of genetic and physical maps and the genome sequences.

Several methods have been developed for construction of physical maps, including hybridization-based [20] and fingerprinting-based methods [21]. Fingerprinting techniques have been widely used in construction of genome-wide physical maps. A number of techniques have been developed to generate fingerprints including the traditional agarose-gel, acrylamide gel, and automated capillary sequencer-based high-information-content fingerprinting methods [21-23]. After comparatively evaluating five fingerprinting methods, it was concluded that the high-information-content fingerprinting method with five enzyme digestion and SNaPshot labeling developed by Luo et al. [22] is the most effective [21]. We report here the construction of a BAC-based physical map of the papaya genome using high-information-content fingerprinting [22]. Contigs on the physical map were aligned with the papaya genome sequence assembly through BAC-end sequences (BES).

## Results

### Fingerprinting and contig map assembly

The papaya BAC library used for fingerprinting was constructed from hermaphrodite 'SunUp', the original transgenic cultivar [11]. This BAC library includes 39,168 BAC clones with the average insert size of 132 kb, providing 13.7× papaya-genome equivalents. All 39,168 BAC clones were fingerprinted and the failed clones were repeated twice, obtaining successful fingerprints for 38,522 BAC clones (98.4% of the BAC library). After excluding the clones with no or small inserts (referred as less than 20 true fragments after editing), and cross-contamination (70% or higher shared fragments for neighboring clones), the remaining 30,824 fingerprints (78.7% of the BAC library) were subjected to contig assembly using the FPC (FingerPrinted Contigs) program.

After automated overlap evaluation and manual review, 26,466 BAC clones were assembled into 963 contigs, while 4,358 clones remained as singletons. The average number of fragments (bands) of each clone was 69.4. Considering the clones with no or small inserts were excluded from contig assembly and the average insert size of the BAC clones used for contig assembly should be higher than 132 kb, the BAC clones mapped on contigs (26,466 BAC clones) represented at least 9.4× genome equivalents of the papaya genome. A total of 571 contigs (59.3% of contigs) contained more than 9 BAC clones with a total of 25,122 BAC clones, accounting for 81.5% of the mapped clones. On average, each contig contains 27.5 BAC clones. The longest contig contains 1571 consensus bands, which is about 0.7% of the total length of the FPC contigs http://www.stardaddy.uga.edu/fpc/WebAGCoL/papaya/WebFPC/. A summary of the papaya FPC map is shown in Table 1.

**Table 1:** Summary of the papaya FPC physical map constructed by high information content fingerprinting

Number of clones with successful fingerprints	30,824
Number of singletons	4,358

Number of clones on contigs	26,466

Number of contigs	963

Contig size distribution	

≥ 200	3
100–199	34
50–99	155
25–49	174
10–24	205
3–9	188
2 clones	204

### Overgo and single-copy probe hybridization

A total of 2,277 overgo probes representing conserved sequences in *Arabidopsis *and genetically mapped Brassica loci were tested against 36,864 papaya BACs. A total of 1,329 overgos (58% of overgos designed) detected positive BACs in the papaya. After eliminating low quality data, the remaining 1,181 overgos were anchored on the papaya FPC map. Among these overgos, 756 (64.0%) hit single contigs; the average number of clones per overgo is 6.0.

Sixteen probes representing single-copy loci of papaya were used to screen the papaya BAC library. A total of 153 positive BAC clones were obtained. These 16 single-copy probes were anchored on the FPC map. Fifteen out of the 16 probes hit single contigs and only the probe of papaya *pistillata *(*PI*) gene hit two contigs, ctg1350 and ctg577. The ctg577 contains only three clones and all of them are PI positive clones. Thus, ctg577 should be part of the ctg1350, but the high stringency setting failed to merge them into a single contig. The probe of *tetA *gene from the transformation vector [12] was placed on ctg972. The ctg972 contains 347 BAC clones, while the total number of consensus bands (CB) is only 557. The detailed analysis of ctg972 is shown in the *Genome Coverage *section. Most clones on ctg972 contain chloroplast sequences. We sequenced 4947 bp upstream and 1713 bp downstream of the flanking regions of *tetA *insertion and the result showed that the 4947 bp upstream fragment shared 98% identity with the papaya chloroplast genome [24]. We conclude that the *tetA *probe was placed on ctg972 due to the border sequence sharing high similarity with the chloroplast genome.

### BAC end sequencing

The entire papaya BAC library of 39,168 BAC clones was end-sequenced. After the first round sequencing, the failed clones were re-arrayed in 384-well plates and re-sequenced. A total of 67,179 BAC end sequences passed the filter after trimming the vector sequences and eliminating the clones with cross-well contamination. The total length of the trimmed sequences is 44,725,370 bp, which accounts for 12% of the papaya genome. The average length of each read is 665.76 bp and the G+C content of the BAC end sequences is 35.0%. Paired ends from 32,397 BAC clones were used to build scaffolds for the whole-genome shotgun sequence assembly and provide anchor points for alignment of the FPC physical map with genome sequences and integration of the genetic and physical maps.

### Integration of physical map, genetic map, and genome sequence

The sequence-tagged SSR genetic map of papaya was used for integrating the genetic and physical maps with the genome sequences [19]. Of the 706 mapped SSR markers, 153 were derived from BAC end sequences (BESs), 466 were designed from shotgun sequence reads, and 87 were developed from assembled shotgun contigs. The BES-derived SSRs directly anchored the FPC contigs to the genetic map. From the 153 BES-derived microsatellites, 122 FPC contigs containing 46,475 consensus bands and 97 shotgun scaffolds covering 132 Mb (35.5% of the papaya genome) were anchored to the genetic map. The 32,397 paired BESs were aligned to the shotgun scaffolds to extend the integrated map. The remaining 553 mapped microsatellites were positioned on shotgun scaffolds by BlastN search. The BAC clones with paired ends covering the mapped microsatellites were used as baits to search the FPC physical map to find the corresponding FPC contigs. The order and orientation of the FPC contigs were examined by aligning on the shotgun scaffolds. The integrated map was further extended with information from the shotgun scaffolds. Additional BAC clones on the anchored shotgun scaffolds were used to search the FPC map for extending the integrated map.

**Figure 1:**
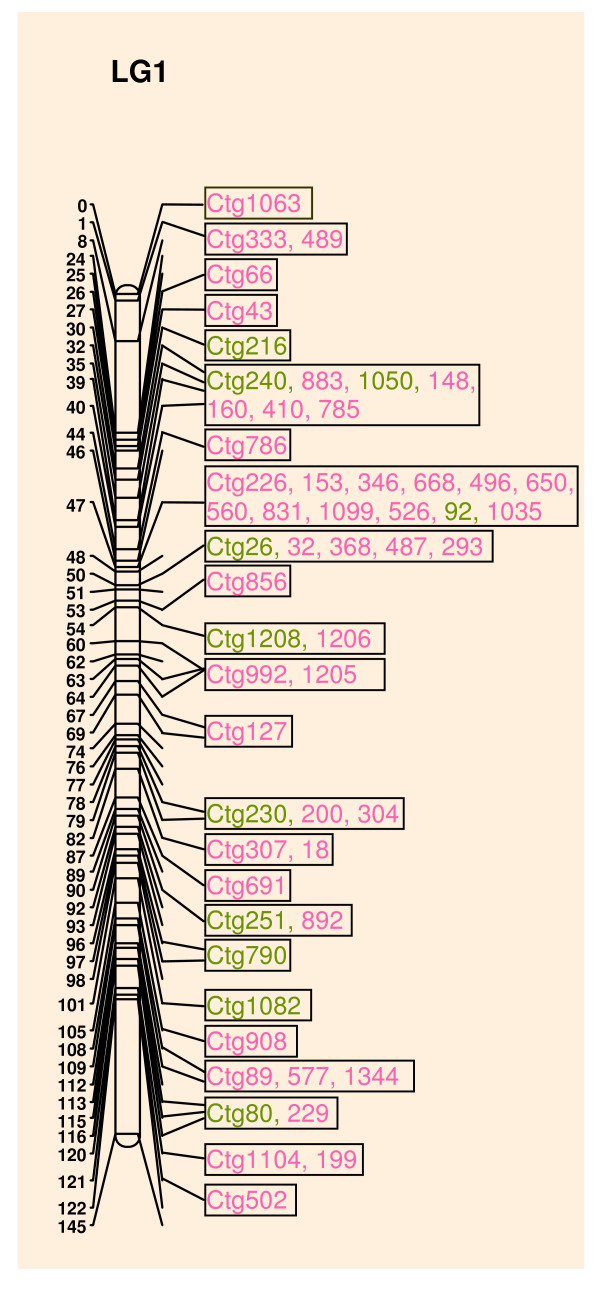
**The integration of FPC contigs and LG1 of the papaya genome**. The numerical scale at the left is the cumulative length of LG1 in centimorgans. The FPC contigs in green color were mapped to LG1 using BES-derived SSRs. The FPC contigs in pink color were mapped to LG1 using shotgun sequence-derived SSRs. The three layers of information, genetic map, FPC contigs, and WGS scaffolds, can be found in the Additional files 2 &3.

The initial integration was conducted by searching the shotgun sequence using BESs of the BAC clones containing the mapped microsatellites to place the shotgun scaffolds and FPC contigs on the genetic map. Detailed information on the initial integration of linkage group 1 (LG1) is showed in Figure 1 and Additional file 1. Among the 77 mapped SSRs on LG1, four were not found in shotgun scaffolds, and three of the four SSRs were MSY-linked, which are not in the female genome. Three SSRs were found in the shotgun scaffolds, but no BAC containing these SSRs was found. Two FPC contigs that were placed on LG1 in the initial integrated map were also mapped onto other linkage groups and were thus removed from our final integrated map. We also used shotgun sequence scaffolds and FPC contigs to validate the initial integration. Overlapping scaffolds and FPC contigs confirmed their order on the genetic map (Additional files 1, 2 &3). For example, FPC contig 1050 connected scaffolds 14 and 140 on LG1. FPC contig 786 corrected the genetic location of the three scaffolds 99, 123, and 39. Eleven genetic loci that had been incorrectly mapped were identified and re-located. Two potential scaffold merges, involving scaffolds 794 and 215, and scaffolds 26 and 286, were recognized. The validated map is shown in Additional file 2. The initial erroneously mapped genetic markers might be the result of segregation distortion and the limited number of individuals used for mapping.

In the final integrated map, 535 (55.6%) FPC contigs containing 21,371 (81.2%) BAC clones and 168,217 consensus bands were anchored on the genetic linkage map. A total of 255 shotgun scaffolds covering 233 Mb were anchored to the genetic map. Overall, 63% of the papaya genome sequences were placed on the genetic map.

Both the FPC contigs and the shotgun scaffolds bridged the gap between linkage groups 8 and 10. Linkage groups 9 and 11 were merged based on fluorescence *in situ *hybridization (FISH) [9]. The estimated physical size of each linkage group is based on the length of each linkage group in centimorgans. Coverage of the integrated map on each linkage group is not even. Linkage group 9+11 showed the highest coverage. Over 90% of this linkage group was covered by anchored shotgun scaffolds and almost 100% was covered by assigned FPC contigs. The least covered linkage group, LG1, is the sex chromosome for which 13% is heterologous male specific region, covering only 43.5% of the estimated physical size (Table 2 and Additional file 1).

**Table 2:** Summary of the integrated genetic and physical map

Genetic Map				Shotgun Sequence			Physical Map				
Linkage Group	No. mapped SSR Markers	Size (cM)	Estimated Physical Size (Mbp)	No. aligned Scaffolds	Length (Mbp)	Coverage	No. aligned FPC Ctgs	No. aligned BACs	Length (CB units)	Estimated Physical Size (Mbp)	Coverage

LG1	77	145.0	50.48	25	21.97	43.52%	56	1892	15682	25.09	49.70%

LG2	70	138.8	48.32	29	27.07	56.02%	63	2345	18877	30.20	62.50%

LG3	116	132.4	46.09	35	29.89	64.85%	68	2796	22187	35.50	77.02%

LG4	57	120.6	41.98	30	22.07	52.57%	44	1840	13946	22.31	53.14%

LG5	63	103.6	36.07	22	24.33	67.45%	48	2287	17704	28.33	78.54%

LG6	106	100.2	34.88	36	29.42	84.35%	65	2819	21458	34.33	98.42%

LG7	59	96.4	33.56	25	21.36	63.65%	62	2050	17602	28.16	83.91%

LG8+10	72	118.9	41.39	29	27.59	66.66%	64	2502	19538	31.26	75.53%

LG9+11	81	91.3	31.78	22	28.70	90.31%	63	2780	20770	33.23	104.56%

LG12	5	21.4	7.45	2	0.96	12.89%	2	60	453	0.72	9.66%

Total	706	1068.6	372	255	233.37	62.73%	535	21371	168217	269.15	72.35%

### Genome coverage

Lengths of the fingerprinted contigs are presented as consensus band (CB) units. To determine average band size and estimate the genome coverage of the fingerprinted physical map, we used information from the draft sequence of the papaya genome. Twenty-two non-overlapping fingerprinted contigs were aligned to the shotgun assembly through BESs (Additional file 4). These 22 FPC contigs of 7,503 CB units cover 12,256,987 bp. We determined that the average band size in our papaya FPC map is about 1.6 kb.

We investigated the organelle genome contamination in our FPC physical map to better estimate genome coverage. Two DNA probes containing sorghum chloroplast *ropB *and *trnK *genes were used to screen the 'SunUp' hermaphrodite papaya BAC library and a total of 568 positive BAC clones were identified, with 179 identified by both probes. We searched for these 568 clones in the papaya FPC map and found 145 of these either formed singletons or were excluded from FPC map construction. Among the remaining 423 clones, 229 were placed on contig 972, 9 on contig 426, and 185 were distributed over 60 other contigs. The BAC end sequences of the 568 positive BAC clones were compared with the papaya chloroplast genome sequence [unpublished data] and 93 BAC clones were identified as having both ends sharing over 90% identity with papaya chloroplast genome sequence. The insert sizes of these 93 BAC clones were determined by Contour-clamped homogeneous electric field (CHEF) electrophoresis. Twelve were confirmed to contain the chloroplast genome sequence by comparing their real measured sizes with the sizes derived from *in silico *analysis of their BESs on the papaya chloroplast genome sequence. Ten out of the 12 BACs were placed on contig 972 and the other two BACs were excluded from the FPC map.

The papaya mitochondrial genome sequence was used as a query to search the papaya BES database. A total of 356 BAC clones was identified with at least one end sharing over 95% identity with the papaya mitochondrial genome sequence. Among them, 144 BACs were verified with both ends containing the papaya mitochondrial genome sequences. Among the 144 BACs, 57 either formed singletons or were excluded from FPC map construction, 26 were placed on contig 867, 24 on contig 524, 15 on contig 1172, 4 on contig 47, 4 on contig 553, and the rest were distributed on 10 other contigs.

The total length of the papaya FPC map is 224,354 CB units and the average number of consensus bands of each contig is 233.0. To estimate the genome coverage of this map, we excluded contigs 972, 426, 867, 524, 1172, 47, and 553, which were confirmed to contain papaya organelle genome sequences. The total length of the remaining contigs was 222,808 CB units and 356.5 Mb (95.8%) of the papaya genome was covered in these FPC contigs. Based on these estimates, we calculated that 269.15 Mb (72.4%) of the papaya genome was anchored to the genetic map (Table 2).

### Genetic recombination

With the shotgun sequences and the FPC contigs both aligned on the genetic map, we examined the relationships between genetic distance and physical distance in the papaya genome. From the papaya genome size of 372 Mb and the total genetic map length of 1068.6 cM, we calculated that the average physical distance per centimorgan of the papaya genome is about 348 kb. We aligned the FPC contigs on the genetic map (Additional file 5). On the integrated map, the ratio between genetic and physical distance varies among chromosomes. Recombination suppressed regions were observed near the center of all major linkage groups (Additional file 5). Hotspots of recombination were found on one end of linkage groups 2, 3, 4, and 5.

**Figure 2:**
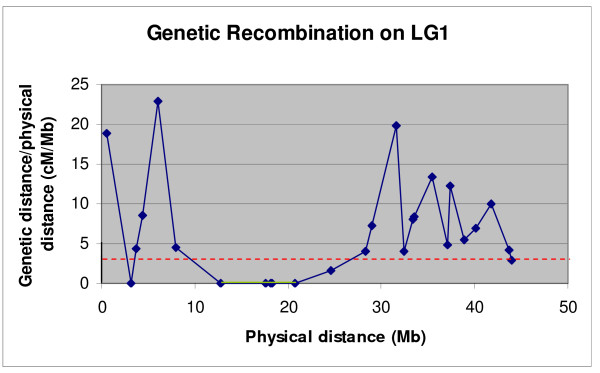
**Variation of recombination rates on LG1 of the papaya integrated map**. The X axis represents the physical distance of integrated contigs in megabases along LG1. The Y axis represents the recombination rates per Mb DNA sequence (cM/Mb). The average rates of recombination across the genome and across the LG1 are at 2.9 cM/Mb as indicated by the dashed red line. The green line highlighted the recombination rate of the MSY region.

The longest linkage group, LG1, contains the MSY. We investigated the variation of the ratio between genetic and physical distance across LG1 (Figure 2). One region with severely suppressed recombination was shown on the center of LG1, and this region is the MSY. The recombination rates at the adjacent regions on both sides of the MSY were gradually recovered as the distance to the MSY increases; dramatically elevated at the locations about 10 Mb away from the MSY to 7-fold of the genome average; and then dropped again. Based on our integrated map, this region covers about 3.9 Mb, which is about 17% of the shotgun sequence aligned on LG1. Since the shotgun sequence aligned the genetic map covered only 43.5% of the entire LG1, we estimated the size of the suppression region to be about 8–9 Mb.

## Discussion

The sequenced papaya SunUp genome is derived from inbred cultivar Sunset with three transgenic insertions, and the papaya genome has no recent genome wide duplication except the ancient triplication event shared by most eudicots [9,25,26]. The genomic organization of papaya is therefore less complicated than that of *Arabidopsis*, which has undergone two rounds of genome wide duplication; or the highly heterozygous poplar genome that has undergone one recent genome wide duplication. Nevertheless, the integration of a BAC-based physical map, genome sequence, and genetic map helped to correct errors and enhance the quality of these resources that are complementary to one another. Integration of these three sets of genomic information provides a valuable resource for comparative genomics, particularly for those researchers who work on the Brassicales [27].

### Genome coverage and representation

The papaya genome size of 372 Mb was estimated by flow cytometry [28]. The papaya draft genome sequence presented an opportunity to verify the genome size estimate by comparing the whole genome shotgun sequence to the fully assembled BAC sequences. However, the only fully sequenced papaya BACs are those on the MSY and its corresponding region of the X chromosome. Since the female papaya genome was sequenced, comparison with the X-specific BACs is valid. Two fully sequenced X BACs, 53E18 and 61H02, consist of 251,868 bp and 168,440 bp, respectively [8]. The whole genome shotgun (WGS) sequence matched 70.3% of the combined 420,308 bp BAC sequences so that the genome size of papaya could be 385 Mb based on the assembled 271 Mb WGS sequence [9]. However, the MSY of papaya is pericentromeric and even the X counterpart has more repetitive sequence than the genome average [29,30], thus the WGS sequence could have been underrepresented in this region. For this reason, the original estimate of 372 Mb might be closer to the actual genome size of papaya.

The papaya physical map was based on the patterns of fragments generated by restriction enzyme digestion. We used the high stringency setting to build up the contig map to minimize false end-merges. Some ends of the contigs might overlap but failed to form larger contigs due to insufficient consensus bands for the high stringency of a minimum 70% overlapping bands. This could result in an overestimation of the genome coverage of the physical map. However, in our assembled FPC map, 4,358 or 14% of clones remained as singletons and 18.5% of the clones located on the small contigs (containing fewer than 9 clones). Compared with the large contigs, singletons and small contigs may be more important for filling gaps in the physical map. Our estimation of the coverage of the papaya physical map was based on the consensus bands and the singletons were not included. Thus, while the genome coverage of the papaya physical map might be overestimated, the scale should be limited.

Accuracy of the genome coverage estimate is also related to the number of contigs in the physical map. The physical map of *Populus *consists of 2,802 contigs, representing 9.4× genome equivalents [31]. The total length of the estimated sizes of the FPC contigs is 577 Mb, about 20% larger than the *Populus *genome size of 485 Mb [31]. In addition to *Populus*, overestimation of genome coverage was also found in the soybean physical map [32]. The soybean physical map consists of 2,905 contigs due to the large number of chromosomes and its highly repetitive genome [32]. The total estimated length of contigs in the soybean physical map is 1,408 Mb, about 26.3% larger than the soybean genome size of 1,115 Mb [32]. In contrast to the maps with large numbers of contigs, in *Arabidopsis*, 27 contigs covered the entire genome except for the centromere, telomere, and nucleolus organization regions (NOR) [20]. In rice, 458 contigs represent about 90.6% of the rice genome [33]. Papaya is a domesticated and inbreeding species and the estimated residual heterozygosity is only about 0.06% for the sequenced gynodioecious cultivar SunUp [9]. The physical map of the papaya genome consists of 963 contigs, which is about 1/3 of the numbers of contigs in the *Populus *and soybean physical maps. Our estimate of the genome coverage of the papaya physical map might be close to the actual percentage.

The level of genome coverage of the physical map relies on the coverage of the underlying BAC library. The BAC library we used for construction of the papaya physical map included 39,168 BAC clones and provided 13.7× genome equivalents. A BAC library usually does not completely cover an entire genome due to non-random distribution of target restriction sites in certain regions of the genome. Moreover, highly repetitive fragments, such as centromeres, telomeres, and NOR regions, are under-represented in the BAC libraries. In papaya, the highly repetitive MSY is a good example of these difficult regions. Extreme gene paucity and a high density of retroelements were observed in the papaya MSY [31]. In our current physical map of the papaya MSY in a separate project to study the evolution of sex chromosomes, the SunUp hermaphrodite BAC library contains nearly all of the 8–9 Mb MSY region (R. Ming, P.H. Moore, A.H. Paterson, J. Jiang, and Q. Yu unpublished data). The physical map of the MSY indicates outstanding coverage of the papaya genome by this BAC library, considering that the centromere of the Y chromosome is likely within the MSY [33].

### Genetic recombination

DAPI (4', 6-diamidino-2-phenylindole) stained papaya chromosomes revealed highly condensed heterochromatin knobs located in the centromeric and pericentromeric regions of all nine pairs of chromosomes [9]. Lengths of the condensed heterochromatin knobs varied among the chromosomes [9]. We examined the genome-wide recombination of papaya based on our integrated map. Consistent with papaya chromosome structure, recombination suppression regions were found at or near the center of all the major linkage groups, suggesting that those regions might be centromeric. It has been demonstrated that the rate of recombination varies among chromosomes, across individual chromosomes, and between different sex types [34-36]. Severe recombination suppression was found in the centromeric and pericentromeric heterochromatin of several species [37-41]. A 53-fold reduction in recombination frequency was found in the left pericentromere of the *Arabidopsis *centromere 1 (CEN1), a 10-fold reduction in the right pericentromere, and 200-fold reduction in the centromeric core [42]. A large quantity of repeats, duplications, and insertions were accumulated in the centromeric and pericentromeric regions, while very few genes were found in these regions [43,44].

The MSY on LG1 is extensively suppressed for recombination. The estimated size of the suppressed region was about 8–9 Mb based on the integrated genetic and physical map, consistent with the 8–9 Mb physical map estimate of the male specific region of the Y chromosome [8]. The physical location of the MSY is in the middle of the Y chromosome based on pachytene FISH images [30], but the genetic map of the MSY was on the upper half of the MSY, likely due to the lower recombination rate on one arm of the Y chromosome [16]. Indeed, pachytene FISH images showed the denser heterochromatic regions on one of the chromosome arms [9]. Fewer markers mapped on this chromosome arm resulting in fewer anchored WGS sequences; it is possible that fewer WGS sequence could be assembled due to the repetitive nature of the heterochromatic regions on this arm. The location of the MSY in Figure 2 is likely distorted for this reason and the physical size of LG1 is underestimated. Interestingly, suppression of recombination spread to adjacent regions of the MSY, but recombination rates recovered gradually first and then increased dramatically at about 10 Mb from the MSY resulting in a hotspot of 20 cM per Mb. This is equivalent to 50 kb per cM, a 7-fold increase compared to the genome wide average of 348 kb per cM. Beyond these two hotspots, the recombination rate declined in the remaining region of both arms with an average about 7.5 cM per Mb at 133 kb per cM or 2.6-fold increase than the genome wide average. Our results demonstrated the dynamics of recombination in a pair of evolving young sex chromosomes that have changed since the initiation of the MSY, and are different from the sudden increase recombination in the small 2.6 Mb and 400 kb pseudo-autosomal regions of the ancient human Y chromosome [45].

It is well known that recombination rate varies along chromosomes. Recombination rates are higher in telomeric regions when compared to centromeric and chromosomal regions. In the human genome, the mean intensity per hotspot is 0.115 cM in telomeric regions, while the mean intensity is 0.070 cM in centromeric regions [46]. Consistently, most recombination suppression regions of papaya were found in the middle of chromosomes and several recombination hotspots were found on one end of LGs 2, 3, 4, and 5. It has been reported that recombination rates were higher in regions with higher gene density [46-48]. Recombination hotspots would be the regions favored by natural selection to produce beneficial allele combinations. To determine whether the recombination hotspots found in papaya are located in gene-riched regions, we would need fine-scale mapping of recombination rates across the genome.

### Potential applications

A genetic linkage map is constructed by placing genetic loci on chromosomes based on recombination frequencies, while the physical map is constructed based on overlapping restriction patterns of large insert BAC clones. Integration of the genetic and physical maps revealed recombination hotspots as well as regions suppressed for recombination. The integrated genetic and physical map allows estimates of physical distances between genetic markers, and provided the framework for assembling the whole genome shotgun sequences. Meanwhile, assembled genome sequence provided precise physical distances between genes and DNA markers in gapless regions. These three genomic resources complement one another and correct errors from each individual source. The combined information enhances the capacity for map-based cloning and identification of underlying genes controlling quantitative traits in papaya.

The draft genome sequence contained 92% of the genes in papaya [9]. To uncover the genes controlling complex traits, we will need to rely on mapping quantitative trait loci (QTLs) and map-based cloning. The integrated map reported here will simplify the process of map-based cloning. A total of 706 SSR markers were integrated with the physical map. Using a subset of these mapped SSR markers to screen segregating populations will allow QTLs to be mapped on intervals of the genetic map, and fine mapping can be carried out using markers from BAC end sequences or WGS sequences within an interval. Most QTLs controlling economically important traits in papaya have not yet been characterized, including those for controlling sugar content, fruit size, shape, and weight, and some complex disease reactions. We expect that our integrated map and genome sequence will expedite the mapping and cloning of target genes and facilitate papaya breeding through marker-assisted selection.

The current draft sequence of the papaya genome represents about 75% of the papaya genome [9]. With rapid progress in next generation sequencing technology, additional papaya whole genome sequencing may be carried out in the foreseeable future. The papaya BAC-based physical map integrated with the genome sequences and genetic map will be an essential resource for closing gaps of any particular genomic region under investigation. In addition, this integrated resource can help identify heterochromatic regions that are difficult to sequence using the current WGS approach, so that a different strategy might be carried out to fill these gaps when effective methods become available. Moreover, the integrated genetic and physical map could also provide a framework to guide the genome sequencing of related species.

A total of 1,181 overgos representing conserved sequences of *Arabidopsis *and genetically mapped *Brassica *loci were anchored on the integrated genetic and physical map and the draft genome sequence of papaya. These overgos are direct links among papaya, *Arabidopsis *and *Brassica *genomes for comparative genomic research among species within the order Brassicales. The overgos were designed from single-copy genes and sequences of *Arabidopsis *and *Brassica*. These anchored overgos further improved the quality of the physical map construction. Along with the FPC contigs and WGS sequences, overgo markers could help identify synteny and rearrangements in target regions of these genomes, particularly in these recently duplicated genomes of *Arabidopsis *and *Brassica *[49-51].

*Carica papaya *is the only species of the genus *Carica*. The small gene pool has limited papaya improvement through traditional breeding. For example, the papaya ringspot virus (PRSV) severely damaged papaya production worldwide. However, development of PRSV-resistant papaya using traditional breeding approaches was not successful due to the lack of PRSV-resistant genes in papaya. *Vasconcellea*, a closely related sister genus of papaya, contains genes resistant to several major virus and fungus diseases, including PRSV. The integrated genetic and physical map can serve as a reference to study gene and genome evolution, to reveal the genetic base of unique ecological adaptations of *Carica *and *Vasconcellea*, and to clone the disease resistance genes from *Vasconcellea *species for papaya improvement.

## Conclusion

We have constructed a BAC-based physical map of *Carica papaya *using high information-content fingerprinting and overgo hybridization of conserved DNA probes from *Arabidopsis *and *Brassica*. This physical map was integrated with a sequence tagged high-density genetic map and a draft genome sequence. The integrated map revealed recombination cold and hot spots in the papaya genome, often associated with known chromosomal features such as the sex chromosomes, centromeres, and telomeres. The recombination rates of the pericentromeric regions are reduced but not completely suppressed. The complete suppression of the 8–9 Mb MSY and the rise and fall of recombination rates in flanking regions indicated the recombination rates in this specialized region evolved over the course of sex chromosome evolution, particularly in these early stages. The integrated map with the draft genome sequence and anchored overgo probes from *Arabidopsis *and *Brassica *is a valuable resource for the plant research community.

## Methods

### BAC DNA isolation and fingerprinting

BAC DNA was isolated with Qiagen R.E.A.L. Prep 96 Plasmid Kit (Qiagen, Valencia, CA) according to the manufacturer's instruction. The purified BAC DNA was fingerprinted according to Luo et al. [19]. BAC DNA was digested simultaneously with five restriction enzymes, *Bam*HI, *Eco*RI, *Xba*I, *Xho*I, and *Hae*III, and labeled with SNaPshot Multiplex System (Applied Biosystems, Foster City, CA). The labeled samples were purified and dissolved in 10 μl of Hi-Di formamide with internal size standard LIZ-500 (Applied Biosystems, Foster City, CA) and labeled restriction fragments were sized with an ABI 3700 DNA Analyzer (Applied Biosystems, Foster City, CA) using POP5 polymer. Fragment size-calling was performed with GeneMapper software (Applied Biosystems, Foster City, CA).

### Physical map construction

Fingerprint profile of each clone collected by the ABI data collection program was processed with the combination of software packages GenoProfiler [52] and FPMiner http://www.bioinforsoft.com/ to remove vector fragments, high frequency fragments, and cross-contaminated clones. The edited sizes data were used for contig assembly with FPC V8.5.3 http://www.agcol.arizona.edu/software/fpc/. Initial assembly was started at stringency of 1 × 10^-45 ^and a tolerance of 0.5 bp, followed by various steps of DQering, end-to-end merge, and manual securitization.

### Genetic map construction

A mapping population of 54 F_2 _individuals derived from variety AU9 crossed by variety SunUp (pollen donor) was used for construction of a high-density genetic map [16]. A high-density genetic map was constructed using 707 markers, including 706 SSR markers and 1 morphological marker, fruit flesh color [16]. The resulting map consisted of 9 major and 3 minor linkage groups and spanned 1068.6 cM with a density at 1.5 cM/marker [16].

### Genome sequencing

A total of 2.8 million whole-genome shotgun (WGS) sequencing reads were generated from variety SunUp by Sanger sequencers [9]. The plastid and mitochondrial genomes were assembled from whole-genome shotgun reads [9]. By using a combination of homology-based and *de novo *methods, a papaya repeat database was constructed [53-55]. After excluding organellar, repetitive, and low-quality reads, a total of 1.6 million reads were assembled into 47,483 contigs containing 271 Mb. With information from the integrated genetic and physical map and the BAC end sequences, a total of 17,764 scaffolds were assembled, covering 370 Mb of the genome.

### BAC end sequencing

Approximately 300 ng–400 ng BAC DNA of each clone was used for BAC end sequencing. The primers used were T7 5'-TAATACGACTCACTATAGGG and M13R 5'-GGATAACAATTTCACACAGG. The sequencing reaction was prepared using ABI BigDye Terminator v3.1 (Applied Biosystems, Foster City, CA) and then analyzed on ABI 3700 and ABI 3730xl DNA Analyzers (Applied Biosystems, Foster City, CA). The sequences were processed with PHRED and trimmed by LUCY. The trimmed BAC end sequences shorter than 100 bp were considered as failed ends and re-arrayed into 384-well plates using Q-PIX (Genetix, Hampshire, UK) and re-sequenced.

### Overgo design and hybridization

Overgo probes were designed using a Microsoft VISUAL BASIC program. Probes containing repetitive sequences were eliminated by comparison with The Institute for Genome Research (TIGR) *Arabidopsis *repeat database. These overgos were radioactively labeled and applied in multiplexed experiments consisting of 576 probes applied with triple redundancy (24 × 24 × 24). Labeled filters were exposed to X-ray films for two weeks. Films were manually scored onto transparencies and scanned into a computer and read by ABBYY FINEREADER 5.0 software. Data were input into BACMan package http://plantgenome.agtec.uga.edu/bacman/ to deconvolute the multiplexed results into individual probes for BAC assignments.

### Screening BAC library

High-density membranes of the papaya BAC library were pre-hybridized in 0.5 M Na_2_HPO_4_, 7% SDS, 1 mM EDTA, 100 ug/ml heat-denatured herring sperm DNA for at least four hours. Probes were labeled by random primer labeling system (RadiPrimerII, GE Healthcare Life Sciences, Piscataway, NJ). The hybridization was performed overnight in 0.5 M Na2HPO4, 7% SDS, 1 mM EDTA, 100 ug/ml heat-denatured herring sperm DNA with ^32^P labeled probes at 65°C. Hybridized membranes were washed twice in 0.5 × SSPE, 0.5% SDS for 10 min at 65°C. The washed membranes were exposed to x-ray film with a pair of intensifying screens at -80°C for 1–6 days depending on the intensity of the signal.

### Mapping BAC end sequences to the draft genome

BAC end sequence reads were trimmed by LUCY and screened for organellar sequences. BAC end sequences with both ends matching with organelle sequences were removed and the rest BAC end sequences were placed on the genome sequences by Arachne based on paired end assembling. The insert sizes of 18,700 BAC clones from the first ligation were estimated at 86 Kb and the rest 20,468 BAC clones from the second ligation contained inserts that averaged at 174 Kb [11].

## Authors' contributions

QY and RM participated in conceiving and coordinating the study, analysis of data, and were the main authors responsible for writing the manuscript. QY, ET, RLS, MJ, JM, PG, and RAA carried out BAC DNA isolation. QY, ET, and RLS conducted BAC fingerprinting. JEB carried out Overgo hybridization. QY, ET, RLS, and SH participated in BES sequencing. QY and MCL participated in FPC contig assembling. PHM, MCL, AHP, and AM contributed to data analyses and interpretation of the results. All authors have read the manuscript and approved it.

## Supplementary Material

Additional file 1 The detailed information of the initial integrated map of linkage group 1. Click here for file

Additional file 2 The detailed information of the final integrated map of linkage group 1. Click here for file

Additional file 3 The detailed information of the integrated genetic and physical map. Click here for file

Additional file 4 Summary of the physical sizes of selected FPC contigs to estimate the average band size in FPC map. Click here for file

Additional file 5 **The FPC contigs were aligned on the genetic map**. The numerical scale at the left of each linkage group is the cumulative length of the LG in centiMorgans. The pink boxes represent FPC contigs. The blue lines highlight the recombination suppression regions and the green lines highlight the recombination hotspots. Click here for file
