# Supplementary material for: A physical map of the papaya genome with integrated genetic map and genome sequence
Source: BMC Genomics. 2009 Aug 7;10:371. doi: 10.1186/1471-2164-10-371 (PMC3224731; doi:10.1186/1471-2164-10-371)
Supplement: Additional file 4 — Summary of the physical sizes of selected FPC contigs to estimate the average band size in FPC map. [file 1471-2164-10-371-S4.doc]

**Additional file 4.** Summary of the physical sizes of selected FPC contigs to estimate the average band size in FPC map.

| Contigs on Physical Map | CB Units* | Physical Size on Shotgun Sequences (bp) |
| --- | --- | --- |
| Ctg43 | 433 | 579,802 |
| Ctg240 | 470 | 870,538 |
| Ctg127 | 452 | 828,244 |
| Ctg691 | 175 | 239,938 |
| Ctg790 | 162 | 409,245 |
| Ctg1082 | 305 | 346,782 |
| Ctg89 | 362 | 531,586 |
| Ctg80 | 347 | 561,872 |
| Ctg190 | 242 | 373,760 |
| Ctg829 | 499 | 938,941 |
| Ctg1295 | 440 | 859,242 |
| Ctg933 | 47 | 61,647 |
| Ctg1333 | 454 | 792,337 |
| Ctg844 | 211 | 254,473 |
| Ctg294 | 203 | 264,320 |
| Ctg767 | 343 | 568,929 |
| Ctg811 | 468 | 739,929 |
| Ctg139 | 316 | 498,460 |
| Ctg991 | 380 | 663,014 |
| Ctg1349 | 298 | 329,359 |
| Ctg290 | 477 | 802,087 |
| Ctg288 | 419 | 742,482 |
| Total | 7,503 | 12,256,987 |

* CB Units: consensus band units.
