# Supplementary material for: A physical map of the papaya genome with integrated genetic map and genome sequence
Source: BMC Genomics. 2009 Aug 7;10:371. doi: 10.1186/1471-2164-10-371 (PMC3224731; doi:10.1186/1471-2164-10-371)
Supplement: Additional file 5 — The FPC contigs were aligned on the genetic map. The numerical scale at the left of each linkage group is the cumulative length of the LG in centiMorgans. The pink boxes represent FPC contigs. The blue lines highlight the recombination suppression regions and the green lines highlight the recombination hotspots. [file 1471-2164-10-371-S5.doc]

0

1

8

24

25

26

27

30

32

35

39

40

44

46

47

48

50

51

53

54

60

62

63

64

67

69

74

76

77

78

79

82

87

89

90

92

93

96

97

98

101

105

108

109

112

113

115

116

120

121

122

145

**LG1**

**(77)**

0

5

7

16

22

24

34

38

45

56

59

61

62

65

66

67

68

69

71

74

77

80

81

82

83

84

97

99

101

103

106

109

111

112

113

115

117

119

125

130

132

134

137

138

139

**LG2**

**(70)**

0

3

4

5

6

9

10

12

20

21

23

25

28

29

30

31

37

41

44

45

47

48

51

54

55

56

57

58

59

60

61

62

63

64

66

67

68

69

70

71

72

73

74

75

76

77

78

80

81

84

87

90

91

93

96

97

98

102

104

105

106

110

116

118

119

120

122

124

125

126

128

129

131

132

**LG3**

**(118)**

Flesh color

0

1

2

3

10

11

16

17

19

21

24

25

31

32

38

40

42

45

46

47

48

49

50

54

56

58

59

62

63

67

68

69

70

74

85

91

99

103

104

107

113

115

118

121

**LG4**

**(57)**

0

13

14

17

18

19

21

22

23

24

25

27

29

30

31

35

36

39

42

44

51

52

54

57

58

59

60

63

64

65

66

68

70

71

77

78

79

80

81

82

84

88

94

96

98

99

104

**LG5**

**(64)**

0

2

3

4

9

10

15

17

21

24

31

33

34

35

36

38

42

43

45

46

47

48

49

50

51

52

55

56

57

58

59

60

61

62

63

64

66

69

71

72

73

74

75

76

77

78

79

80

81

82

83

84

85

86

88

90

94

100

**LG6**

**(106)**

0

7

9

16

18

21

24

28

31

33

37

38

41

43

47

49

50

51

52

53

56

57

58

59

60

61

67

70

71

73

75

76

80

81

82

85

90

91

92

93

95

96

**LG7**

**(59)**

0

2

7

12

15

20

25

31

34

37

40

43

44

45

46

47

48

49

50

51

53

57

60

61

62

68

81

86

92

**LG8**

**(60)**

0

3

5

6

7

8

10

15

16

17

18

19

20

21

24

26

27

28

29

30

31

32

33

34

35

37

38

39

41

43

44

45

46

50

52

58

64

**LG9**

**(75)**

0

8

17

19

21

22

26

27

**LG10**

**(12)**

0

5

7

15

26

27

**LG11**

**(8)**

0

6

7

12

21

**LG12**

**(5)**
